# MYCN promotes neuroblastoma malignancy by establishing a regulatory circuit with transcription factor AP4

**DOI:** 10.18632/oncotarget.10709

**Published:** 2016-07-19

**Authors:** Chengyuan Xue, Denise M.T. Yu, Samuele Gherardi, Jessica Koach, Giorgio Milazzo, Laura Gamble, Bing Liu, Emanuele Valli, Amanda J. Russell, Wendy B. London, Tao Liu, Belamy B. Cheung, Glenn M. Marshall, Giovanni Perini, Michelle Haber, Murray D. Norris

**Affiliations:** ^1^ Children's Cancer Institute, Lowy Cancer Research Centre, University of New South Wales, Sydney, Australia; ^2^ Department of Pharmacy and Biotechnology, University of Bologna, Bologna, Italy; ^3^ Boston Children's Hospital and Dana-Farber Cancer Institute, Harvard Medical School, Boston, MA, USA; ^4^ Kids Cancer Centre, Sydney Children's Hospital, Sydney, Australia; ^5^ CIRI Health Sciences and Technologies University of Bologna, Bologna, Italy; ^6^ University of New South Wales Centre for Childhood Cancer Research, Sydney, Australia

**Keywords:** neuroblastoma, cancer, MYCN oncogene, TFAP4, cell migration

## Abstract

Amplification of the *MYCN* oncogene, a member of the MYC family of transcriptional regulators, is one of the most powerful prognostic markers identified for poor outcome in neuroblastoma, the most common extracranial solid cancer in childhood. While MYCN has been established as a key driver of malignancy in neuroblastoma, the underlying molecular mechanisms are poorly understood. Transcription factor activating enhancer binding protein-4 (TFAP4) has been reported to be a direct transcriptional target of MYC. We show for the first time that high expression of *TFAP4* in primary neuroblastoma patients is associated with poor clinical outcome. siRNA-mediated suppression of *TFAP4* in MYCN-expressing neuroblastoma cells led to inhibition of cell proliferation and migration. Chromatin immunoprecipitation assay demonstrated that *TFAP4* expression is positively regulated by MYCN. Microarray analysis identified genes regulated by both MYCN and TFAP4 in neuroblastoma cells, including Phosphoribosyl-pyrophosphate synthetase-2 (PRPS2) and Syndecan-1 (SDC1), which are involved in cancer cell proliferation and metastasis. Overall this study suggests a regulatory circuit in which MYCN by elevating TFAP4 expression, cooperates with it to control a specific set of genes involved in tumor progression. These findings highlight the existence of a MYCN-TFAP4 axis in MYCN-driven neuroblastoma as well as identifying potential therapeutic targets for aggressive forms of this disease.

## INTRODUCTION

The *MYC* family of proto-oncogenes play important roles as transcriptional regulators in vital cellular functions [1]. The most well-characterised member of the family, MYC, is frequently deregulated in adult cancers [2, 3]. In neuroblastoma, the most common extracranial solid tumor of childhood accounting for approximately 15% of all childhood cancer related deaths, amplification of the *MYCN* oncogene in tumors represents one of the most powerful prognostic markers yet identified for this malignancy [4]. Although *MYCN* amplification and consequent overexpression has been established as a key driver of malignancy in *MYCN*-amplified high-risk neuroblastoma, the exact mechanisms by which MYCN contributes to the aggressive phenotype of neuroblastoma remain largely unknown [4]. Developing a greater understanding of some of the underlying mechanisms of MYCN-mediated neuroblastoma progression is important for identifying genes responsible for tumor progression as well as potential molecular therapeutic targets.

TFAP4 is a member of the basic helix-loop-helix transcription factors that recognize the E-box sequence CAGCTG in the promoters of target genes [5], and has been shown to be a direct transcription target of MYC [6]. Recently, there has been increasing evidence that TFAP4 plays important roles in human cancer development and progression and in particular, it has been found to be a direct inducer of epithelial–mesenchymal transition (EMT) that contributes to metastatic processes in colorectal cancer [7]. In addition, elevated TFAP4 expression significantly correlates with tumor progression and poor prognosis in a number of malignancies, including colorectal cancer [7, 8], gastric cancer [9] and non-small cell lung cancer [10]. Conversely, down-regulation of this transcription factor blocks proliferation of human gastric cancer cells [9] and inhibits metastasis of colorectal cancer cells in mice [7]. Therefore as a MYC transcriptional target, TFAP4 may play a critical role in cancer cells in concert with deregulated MYC, by coordinating the expression of specific genes that are essential for tumor progression. In this study, the relevance of TFAP4 in the context of MYCN-driven neuroblastoma was investigated.

## RESULTS

### *TFAP4* is directly regulated by MYCN

**Figure 1:**
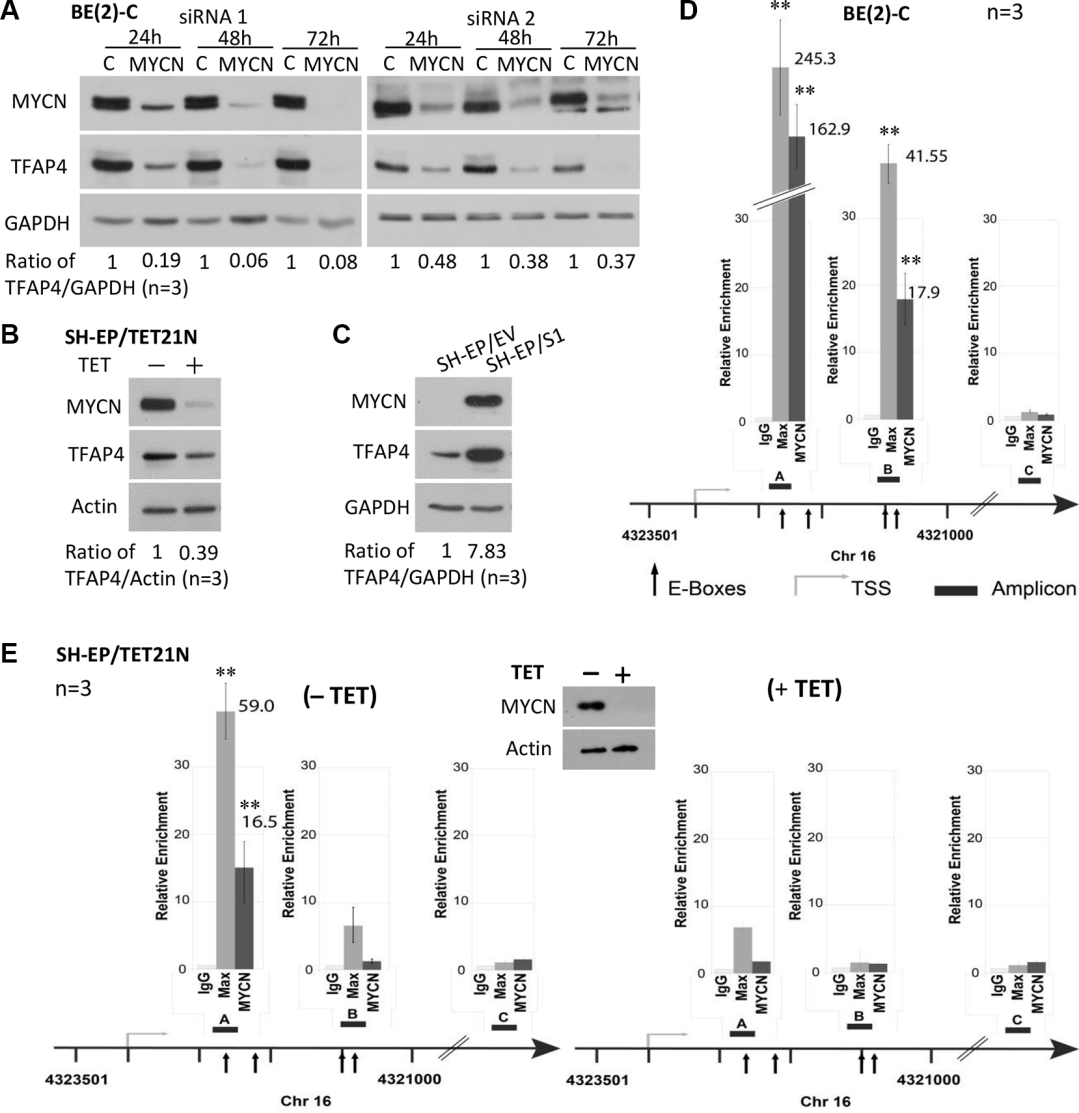
TFAP4 is regulated by MYCN in neuroblastoma cells Suppression of *MYCN* resulted in down-regulation of TFAP4 in BE(2)-C cells (**A**). TFAP4 expression levels paralleled MYCN expression in SH-EP/TET21/N cells (MYCN Tet-Off system, 24 hours) (**B**) and in neuroblastoma SH-EP/S1 cells constitutively expressing exogenous *MYCN* compared with SH-EP/EV controls (**C**). GAPDH or Actin served as protein loading controls on Western blot. Quantitative ChIP assays in BE(2)-C cells (**D**) and SH-EP/TET21/N cells expressing MYCN (E) or treated with tetracycline (TET) for 48 h (**E**) demonstrated that MYCN directly binds to two E-box sites (amp A and B) located in the first intron of the *TFAP4* gene, but not the control region (amp C). Western blot confirmed repression of MYCN expression with tetracycline treatment (E, inset). Mean ± SE (*n* = 3). amp, amplicon; TSS, transcription start site. **P < 0.01.

Since TFAP4 has been reported to be a direct transcriptional target of MYC in adult breast cancer cells [6], we investigated whether a similar relationship exists between TFAP4 and MYCN in neuroblastoma by performing expression analysis following knockdown of *MYCN* in *MYCN*-amplified neuroblastoma cell lines BE(2)-C, CHP-134 and IMR-32. We observed that TFAP4 protein levels were markedly downregulated following *MYCN* depletion (Figure 1A, Supplementary Figure S1A). Similar results were demonstrated in *MYCN*-inducible SH-EP/TET21/N human neuroblastoma cells following tetracycline treatment (Figure 1B, Supplementary Figure S1B). Additionally, increased MYCN expression was found to correlate with increased TFAP4 protein and RNA expression in SH-EP/S1 cells stably overexpressing *MYCN* compared to SH-EP/EV controls (Figure 1C, Supplementary Figure S1B).

To assess whether MYCN is a direct transcriptional regulator of TFAP4, quantitative chromatin immunoprecipitation (qChIP) assays were performed in BE(2)-C and SH-EP/TET21/N cells. MYC has been reported to bind to three of four canonical E-boxes (CACGTG) in the first intron of *TFAP4* [6]. Using MYCN and Max antibodies, we confirmed that both MYCN and Max strongly bound to these E-box motifs (amp A+B), but not to a control region (amp C) located in intron 6 of *TFAP4* (Figure 1D–1E). Interestingly, differences in the relative ChIP enrichments observed between BE(2)-C and SH-EP/TET21/N cells reflect the intrinsic level of MYCN expressed in these cells which is markedly higher in BE(2)-C than SH-EP/TET21/N, even when the latter are induced to express MYCN. The fact that MYCN binding is consistently observed in both cell lines for E-box A but only in BE(2)-C for E-box B may be explained by the variability of fragmented DNA size used for the ChIP assays. Nevertheless, these observations are consistent with the accepted notion that MYC activity is exerted nearby the transcription start site, and that maximal binding of MYC to promoters occurs at the transcription start site and fades with distal E-box elements. Specificity of the MYCN binding to the E-box motifs was supported by a striking reduction of MYCN binding to DNA when MYCN expression was repressed (Figure 1E). Collectively, these data indicate that *TFAP4* is a direct transcriptional target of MYCN in neuroblastoma.

### TFAP4 promotes cell growth in neuroblastoma

**Figure 2:**
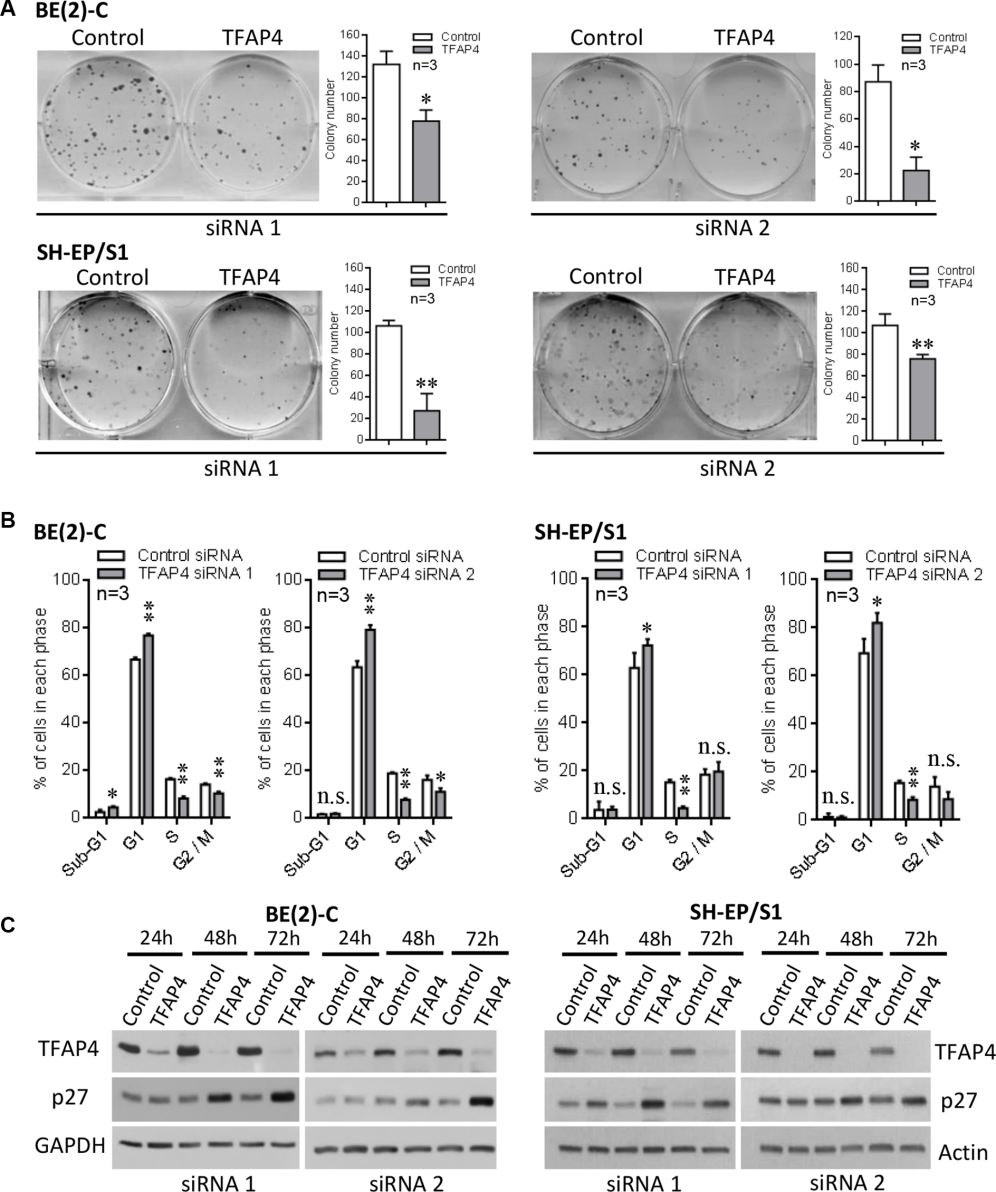
Inhibition of neuroblastoma cell growth following knockdown of *TFAP4* Knockdown of *TFAP4* reduced colony forming ability in *MYCN*-amplified BE(2)-C and *MYCN*-overexpressing SH-EP/S1 cells (**A**). Cell cycle analysis showed strong G1/S arrest 48 h after *TFAP4* depletion (**B**). Western blots showed increased p27 in *TFAP4*-depleted BE(2)-C and SH-EP/S1 (**C**). *P < 0.05, **P < 0.01, n.s.- not statistically significant.

We next investigated whether TFAP4 promotes cell growth in *MYCN*-amplified neuroblastoma. We observed that knockdown of *TFAP4* in both *MYCN*-amplified neuroblastoma BE(2)-C cells, and SH-EP/S1 neuroblastoma cells (stably overexpressing MYCN) resulted in a marked decrease in colony forming ability of these cells (Figure 2A), and cell cycle arrest at G1 and S phases (Figure 2B, Supplementary Figure S1C), while BE(2)-C cells also demonstrated a decrease in the cell population at G2/M phase. We next investigated potential alterations in cyclin-dependent kinase inhibitor levels and a marked upregulation of p27^Kip1^ (Cdkn1b) was observed in both cell lines (Figure 2C). These results show that suppression of *TFAP4* leads to an increase in cyclin-dependent kinase inhibitor levels, which may contribute to the reduced growth phenotype of neuroblastoma cells.

### TFAP4 is required for cell migration in MYCN-overexpressing neuroblastoma cells

**Figure 3:**
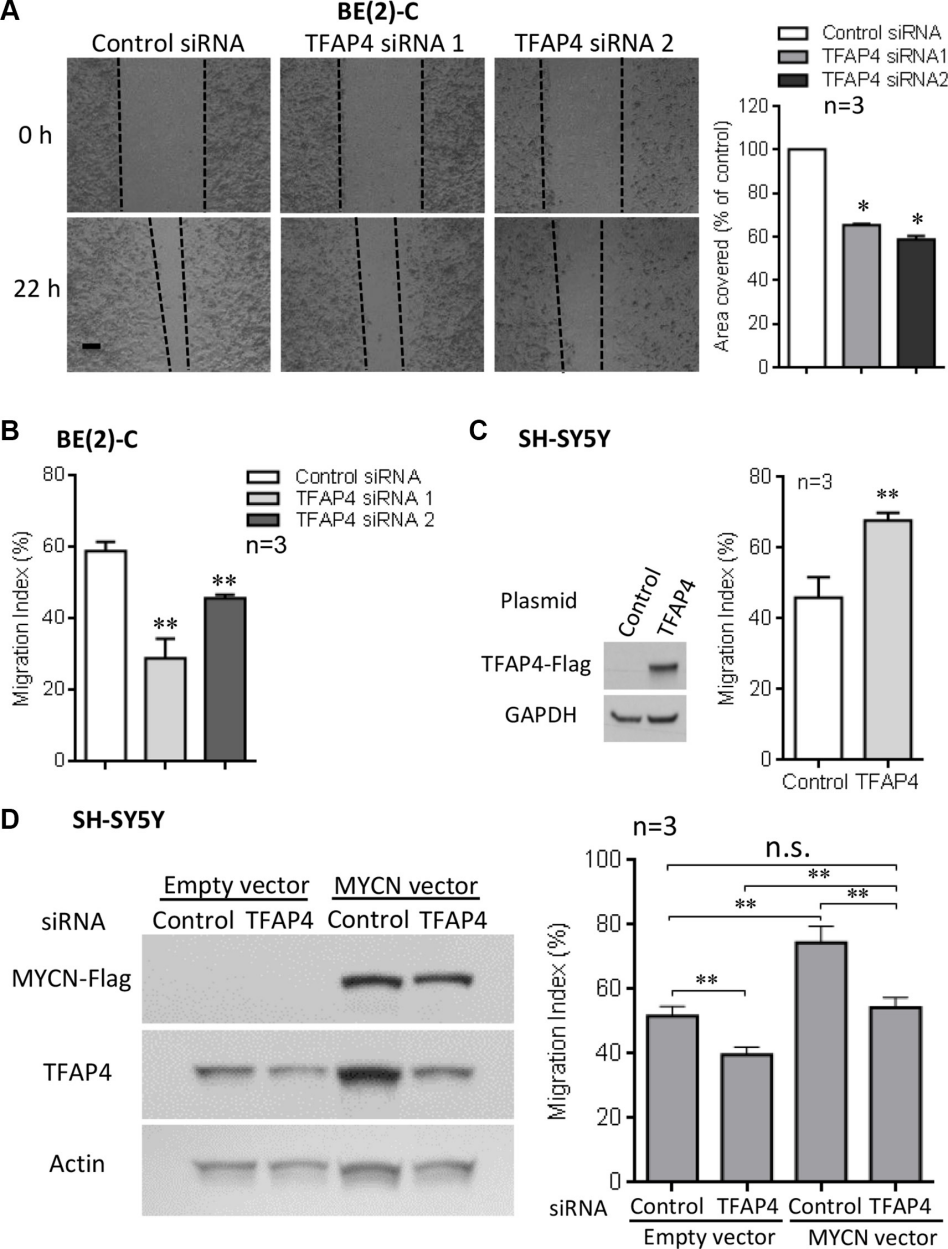
TFAP4 is required for neuroblastoma cell migration Suppression of *TFAP4* in BE(2)-C decreased cell motility in wound closure assays (**A**) (Scale bar 100μm). *TFAP4* suppression decreased transwell migration (**B**). Overexpression of *TFAP4* in *MYCN*-non-amplified SH-SY5Y cells increased migration (**C**). Transient overexpression of *MYCN* in SH-SY5Y cells increased migration, and depletion of *TFAP4* abrogated this effect (**D**). Western blots demonstrated successful transfection of *MYCN* plasmid, *TFAP4* plasmid or *TFAP4* siRNA (C, D). Mean ± SE (*n* = 3). *P < 0.05, **P < 0.01.

In addition to a growth inhibitory phenotype, we observed that *TFAP4* knockdown in BE(2)-C cells also led to reduced cell motility compared with control siRNA transfected cells, as measured by wound closure (Figure 3A) and transwell migration assays (Figure 3B). To confirm the role of TFAP4 in neuroblastoma cell migration, we next performed cell migration assays after transiently overexpressing *TFAP4* in *MYCN* non-amplified cell lines (SH-SY5Y and SK-N-FI), which express relatively low levels of both MYCN and TFAP4. In both SH-SY5Y and SK-N-FI, overexpression of *TFAP4* demonstrated enhanced migratory ability compared to empty vector-transfected controls (Figure 3C, Supplementary Figure S2A). To further evaluate the importance of TFAP4 in MYCN-driven neuroblastoma migration, SH-SY5Y neuroblastoma cells were transiently transfected initially with a *MYCN*-overexpressing construct, followed 24 hours later by *TFAP4* or control siRNA. While an increase in migration compared to controls was observed with *MYCN* overexpression, *TFAP4* depletion abrogated this effect, restoring migration to similar levels as control siRNA-treated cells (Figure 3D). Taken together, these data suggest that TFAP4 is required for the MYCN-driven neuroblastoma migration phenotype.

### *TFAP4* gene expression is associated with poor clinical outcome in primary neuroblastoma

**Figure 4:**
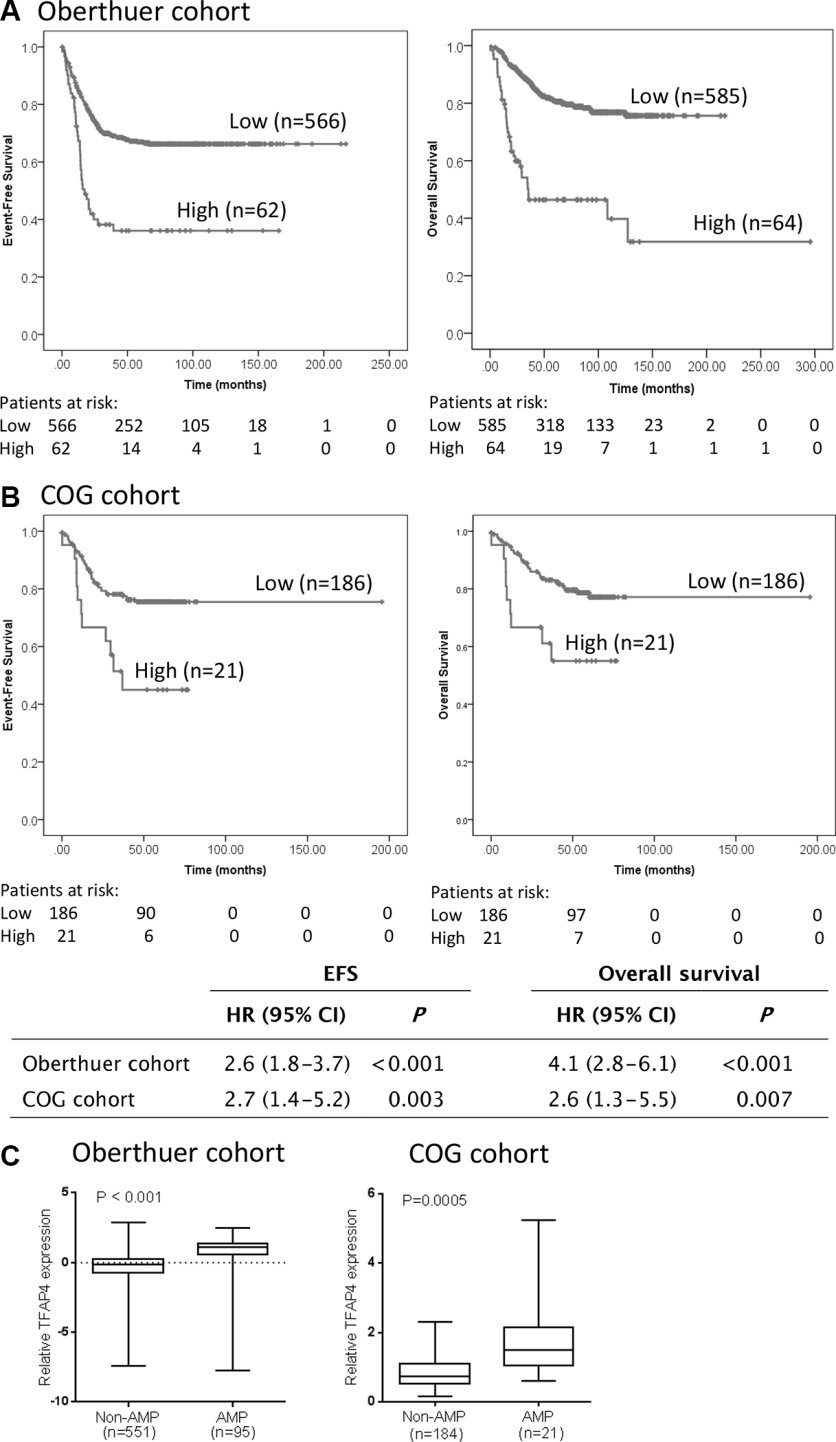
*TFAP4* gene expression is prognostic for poor outcome in primary neuroblastoma Kaplan–Meier curves for event-free survival (EFS) and overall survival (OS) from (**A**) Oberthuer cohort (*n* = 649) and (**B**) COG cohort (*n* = 208), dichotomized around the upper decile. (**C**) *TFAP4* expression levels were significantly higher in *MYCN*-amplified compared to non-*MYCN*-amplified tumors.

To determine whether *TFAP4* has prognostic significance in primary neuroblastoma, we analysed *TFAP4* gene expression and its association with clinical outcome in an expression array dataset from a prospectively accrued primary neuroblastoma cohort of 649 patients (Oberthuer cohort) [11]. *TFAP4* expression was strongly associated with both poor event-free survival (EFS) and overall survival (OS) when samples were dichotomised using the upper decile as a cut point (Figure 4A). These results were validated in another cohort of 208 patient samples from Children's Oncology Group (COG) using qPCR-based methodology (Figure 4B). However, multivariate analysis, taking into account patient age, stage and *MYCN* status, showed that TFAP4 was not an independent prognostic marker (data not shown), suggesting its upregulation could be largely dependent on *MYCN* overexpression. Concordantly, *TFAP4* gene expression levels were significantly higher in *MYCN*-amplified tumors by comparison with non-*MYCN* amplified tumors (Figure 4C) and positively correlated with *MYCN* expression in both datasets (Supplementary Figure S2B).

### Identification of genes regulated by MYCN through TFAP4

Both MYCN and TFAP4 are known to be involved in regulation of important cell biological processes such as proliferation and migration [7, 12]. Thus, in order to identify a subset of genes that may be responsible for mediating the effects of MYCN and TFAP4, we performed expression array analyses of differential gene expression in BE(2)-C 30 h post-transfection with either *MYCN* or *TFAP4* siRNA (GEO repository accession no. GSE74626). This relatively early timepoint was selected for gene expression analysis to identify direct target genes of both MYCN and TFAP4. Using a two-fold cut-off, the results showed that *MYCN* depletion resulted in downregulation of 98 genes out of a total of 20066 genes (0.49%), and three of these genes (3.06%), *PRPS2*, *SDC1* and *SLC7A6,* were also downregulated with *TFAP4* depletion (Supplementary Figure S2C), indicating that these genes were positively regulated by both MYCN and TFAP4. *MYCN* depletion also resulted in upregulation of 127 genes (0.63%), with 12 of these genes (9.45%) also upregulated following *TFAP4* depletion, indicating that these genes were negatively regulated by the two transcription factors. The number of genes identified using a two-fold cut-off following *MYCN* siRNA-mediated inhibition 30 h post-transfection, was similar to that found in a previous study in neuroblastoma using this time point [13]. In addition, no genes were found to be both upregulated by *MYCN* depletion and downregulated by *TFAP4* depletion, or vice versa (Supplementary Figure S2C), which meant that TFAP4 and MYCN did not regulate gene expression in opposing directions.

**Figure 5:**
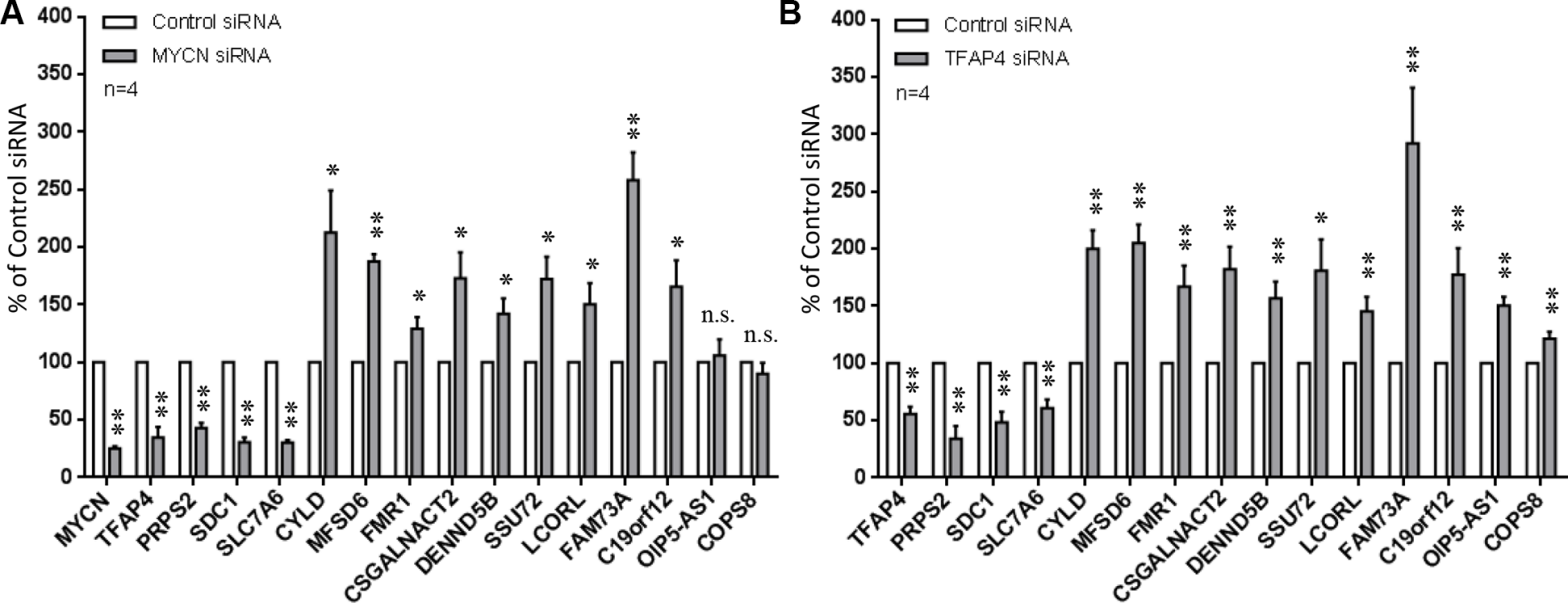
Confirmation of differentially expressed genes from microarray Gene expression was measured by qPCR 48 h after depletion of *MYCN* (**A**) or *TFAP4* (**B**) in BE(2)-C cells. Mean ± S.E. (four independent transfections with two siRNAs to either *MYCN* or *TFAP4)*. *P < 0.05, **P < 0.01.

To confirm differential expression of these genes (Table 1), qPCR was performed on *MYCN*- and *TFAP4*-depleted BE(2)-C cells except for the uncharacterized gene *LOC100499177* (due to unavailability of TaqMan^®^ assay). Loss of *MYCN* correlated with decreased expression of *TFAP4* as well as the three positively regulated genes *PRPS2*, *SDC1* and *SLC7A6,* and increased expression of the negatively regulated genes *CYLD* (*Cylindromatosis*), *MFSD6*, *FMR1*, *CSGALNACT2*, *DENND5B*, *SSU72*, *LCORL*, *FAM73A* and *C19orf12* at 48 h post-transfection (Table 1). Expression of *OIP5-AS1* was slightly increased after *MYCN* depletion but not statistically significant, and expression of *COPS8* was unchanged (Figure 5A). For *TFAP4* depletion, the qPCR data for every single gene tested was consistent with the microarray findings (Figure 5B). Thus, 12 out of the 15 differentially regulated genes identified in the expression array analyses were verified by qPCR.

**Table 1:** Genes showing two-fold differential expression 30 hours after depletion of *MYCN* or *TFAP4* by siRNA knockdown in BE(2)-C neuroblastoma cells

Gene symbol	Function
Genes downregulated by *MYCN* & *TFAP4* siRNA	
*PRPS2*	Synthesis of purines and pyrimidines
*SDC1*	Cell proliferation, migration and cell-matrix interactions
*SLC7A6*	Amino acid transport
Genes upregulated by *MYCN* and *TFAP4* siRNA	
*CYLD*	Deubiquitination, inhibition of NF-kappa-B and HDAC6
*MFSD6*	Transmembrane transport of small solutes
*FMR1*	Translational repression
*CSGALNACT2*	Elongation during chondroitin sulfate synthesis
*DENND5B*	Promotes the exchange of GDP to GTP
*SSU72*	Dephosphorylation of RNA polymerase II C-terminal domain
*LCORL*	Spermatogenesis, association with adult height
*FAM73A*	Uncharacterized, likely responsible for obesity
*C19orf12*	Uncharacterized, mutation found in neurodegeneration
*OIP5-AS1*	Antisense RNA to *OIP5* gene
*COPS8*	positive regulation of E3 ubiquitin ligases
*LOC100499177*(uncharacterized gene)	Uncharacterized

### *PRPS2* and *SDC1* are directly regulated by MYCN and TFAP4

**Figure 6:**
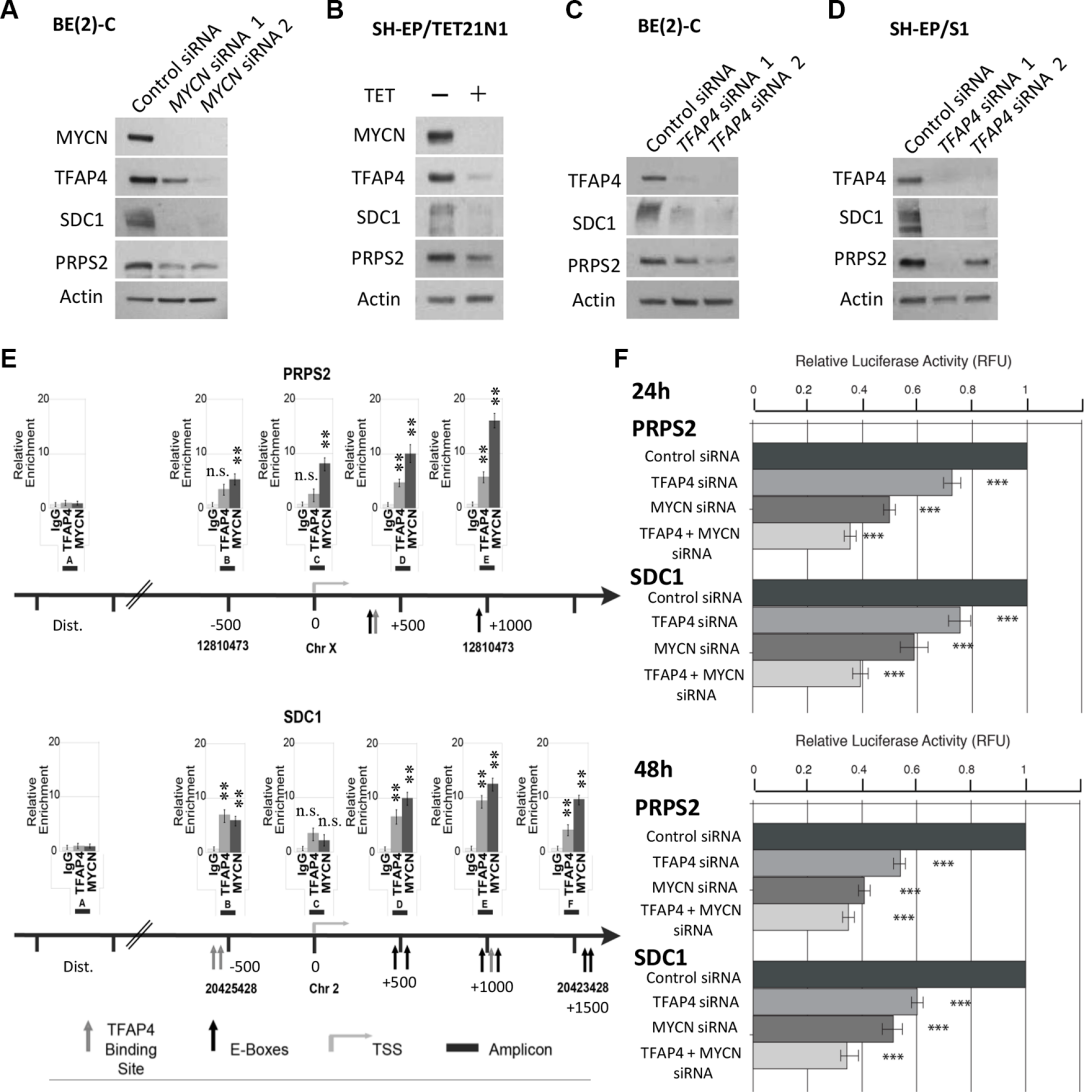
PRPS2 and SDC1 are direct transcriptional targets of MYCN and TFAP4 in neuroblastoma (**A**–**D**) Western blots of PRPS2 and SDC1 expression in BE(2)-C and SH-EP/S1 48 h after suppression of *MYCN* or *TFAP4* or 72 h after suppression of *MYCN* expression in SH-EP/TET21/N cells (*n* ≥ 2). (**E**) Quantitative ChIP assays on BE(2)-C cells. Fold enrichment is relative to the preimmune serum. Mean ± S.E. (*n* = 5) in which each region was amplified by qPCR in triplicate. Bent arrow: transcriptional start site (TSS); Dist. (Distal region); grey arrow: TFAP4 binding site; black arrow: E-box (MYCN binding site); black boxes: amplicons indicated with a capital letter. The chromosome and coordinates (bp) are provided. (**F**) Luciferase activity was determined following transfection of reporter constructs into BE(2)-C transfected with *TFAP4* and/or *MYCN* siRNA, or control siRNA. ***P < 0.001.

It has been demonstrated in the literature that both MYCN and TFAP4 are effective at both activation and repression of target genes [7, 14]. Although most of our identified candidate genes were repressed by MYCN and TFAP4 we decided to focus on the positively regulated genes for essentially two reasons. Firstly, MYCN negative regulation of target genes is not well defined and occurs either through interaction with other accessory transcription factors or through secondary mechanisms, as we have described [15]. Secondly, and more importantly, our interest is directed towards the identification of genes whose inhibition makes them potentially valuable targets for development of new anticancer therapies. We therefore chose to first examine the roles of *PRPS2* and *SDC1* that have been associated with rapid tumor progression and metastasis in certain types of human cancers other than neuroblastoma [16–18]. At the protein level, suppression of *MYCN* resulted in dramatic decreases in expression of SDC1 and PRPS2 in neuroblastoma BE(2)-C and SH-EP/TET21/N cells (Figure 6A–6B). Consistently, knockdown of *TFAP4* by siRNA corresponded with decreased SDC1 and PRPS2 expression (Figure 6C–6D).

Examination of the promoter regions of PRPS2 and SDC1 revealed a number of MYCN and TFAP4 binding sites in close vicinity to one another (Figure 6E), however no interaction between MYCN and TFAP4 was detected by co-immunoprecipitation (data not shown), suggesting that *PRPS2* and *SDC1* may be cooperatively regulated by both MYCN and TFAP4. Subsequent ChIP assays in BE(2)-C confirmed that both MYCN and TFAP4 bound to the promoters of *PRPS2* and *SDC1* in the vicinity of their respective E-box binding motifs (CACGTG for MYCN and CAGCTG for TFAP4), but not the control regions (Figure 6E). Luciferase reporter assays also showed that activity for both *PRPS2* and *SDC1* was significantly decreased with *TFAP4* or *MYCN* depletion, and further decreased with their combined depletion (Figure 6F). Taken together, these data indicate that both *PRPS2* and *SDC1* are co-operatively regulated by both MYCN and TFAP4 in neuroblastoma cells.

### PRPS2 and SDC1 are effectors of MYCN-mediated neuroblastoma progression

**Figure 7:**
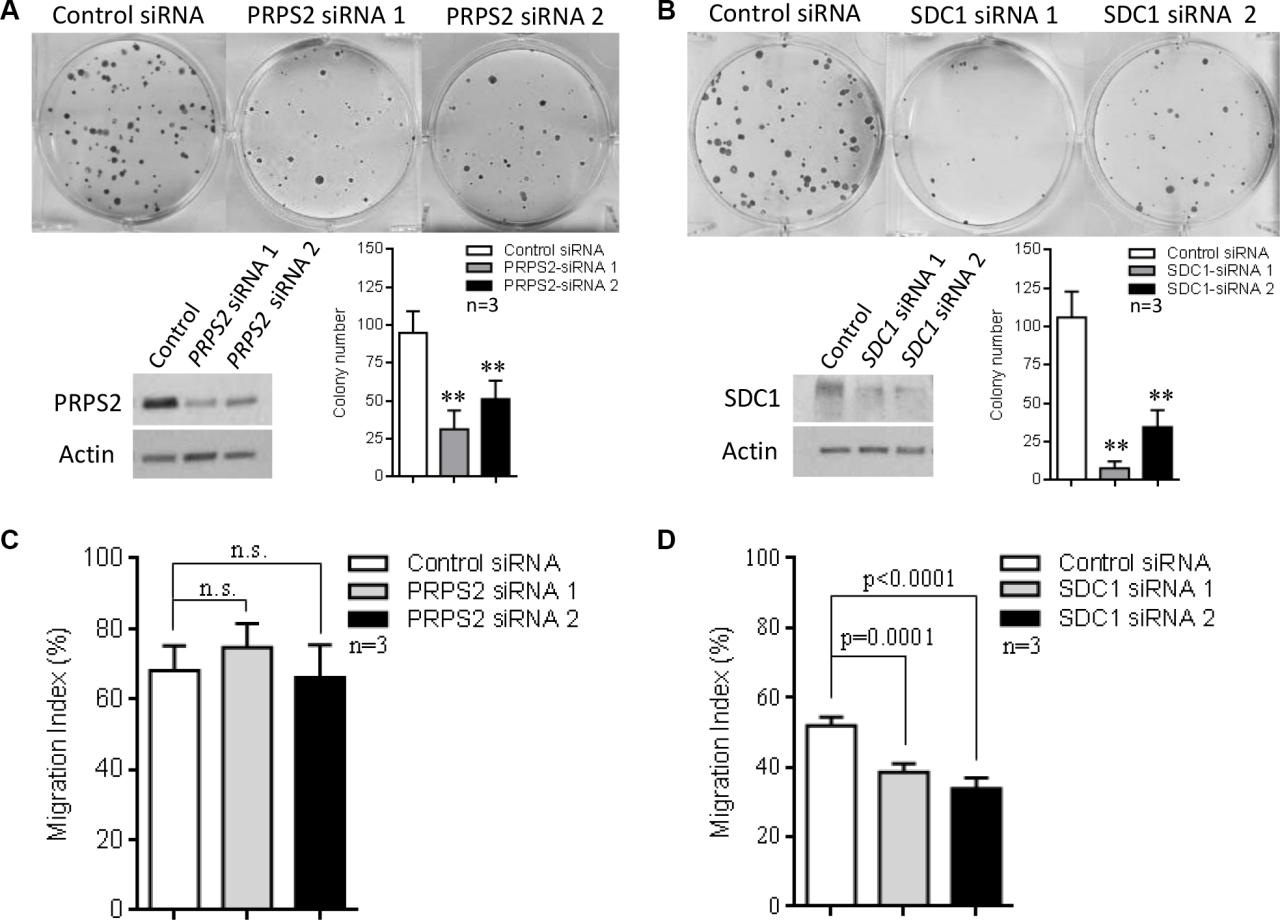
The role of PRPS2 and SDC1 in neuroblastoma progression (**A**–**B**) Suppression of *PRPS2* or *SDC1* resulted in reduction of colony forming ability in BE(2)-C cells. (**C**–**D**) *SDC1* suppression significantly decreased migration, while *PRPS2* suppression showed no significant effect. Mean ± SD (*n* = 3), **P < 0.01.

Knockdown of *PRPS2* or *SDC1* led to a marked decrease in colony forming ability of BE(2)-C cells (Figure 7A–7B). Since SDC1 has been reported to mediate tumor growth and migration [19], we further investigated its role in the migration of BE(2)-C cells. We observed that suppression of *SDC1* significantly decreased transwell migration of these cells, while repression of *PRPS2* showed no effect on migration (Figure 7C–7D). Finally, we analyzed *PRPS2* and *SDC1* expression and their association with clinical outcome in the 649 neuroblastoma expression array dataset. Kaplan–Meier survival analysis showed that high levels of either *PRPS2* or *SDC1* expression were strongly associated with poor EFS and OS (Supplementary Figure S3A–S3B), suggesting that both these genes may play important roles in MYCN-driven neuroblastoma cell proliferation and tumor progression. The prognostic significance of these two genes was also retained when the analysis was confined to the subset of *MYCN*-amplified tumours (Supplementary Figure S3C) further suggesting a contribution to the MYCN-driven malignant phenotype. In addition, in the same dataset, we found that high levels of expression of *SLC7A6*, another gene positively regulated by MYCN and TFAP4, were significantly associated with poor EFS and OS, but high levels of expression of *CYLD,* a gene repressed by MYCN and TFAP4, were associated with good EFS and OS (Supplementary Figure S4A–S4B).

### The role of TFAP4 in the regulation of EMT in neuroblastoma

TFAP4 has been shown to be a mediator of epithelial–mesenchymal transition (EMT) in colorectal cancer [7]. To investigate the role of this transcription factor in inducing an EMT phenotype in *MYCN*-amplified neuroblastoma, we investigated a panel of eleven critical EMT-associated genes, including *ACTN4*, *KRT8*, *MYH9*, *ROCK1*, *TCF3*, *VIM* (*Vimentin*), *ZEB1*, *TWIST* (*TWIST1*), *SNAIL* (*SNAI1*), *CDH1* (*E-cadherin*) and *CDH2* (*N-cadherin*). Initially, we referred to our gene expression profiling data on BE(2)-C cells 30 h after depletion of *TFAP4* with two different siRNAs to explore whether there was a significant change of expression of these genes. We found only subtle differential expression of these genes, with no single gene's expression change being greater than 1.5-fold following siRNA-mediated *TFAP4* knockdown. Following this, we performed transient overexpression of *TFAP4* in two neuroblastoma cell lines, SH-SY5Y and SK-N-FI which have relatively low levels of endogenous TFAP4. Overexpression of *TFAP4* was confirmed by Western blot (data not shown) and RNA expression of the EMT-associated genes was measured by qPCR at 24 and 48 hours post transfection (Supplementary Figure S5). We found that *CDH1* was significantly upregulated by overexpression of *TFAP4* at 24 and 48 hours in both SH-SY5Y and SK-N-FI cell lines. *SNAIL* was significantly upregulated at 24 h in SH-SY5Y cells but not in SK-N-FI cells, although a trend of increasing expression was observed in SK-N-FI cells. *KRT8* and *ZEB1* expression were reduced at 24 h in response to elevated TFAP4 levels in SH-SY5Y cells. *KRT8* expression was also lower at 24 h in SK-N-FI cells but not statistically significant. The remaining genes displayed no significant change in the presence of overexpressed *TFAP4*.

## DISCUSSION

In this study, we have demonstrated that *TFAP4* is a direct transcriptional target of MYCN in neuroblastoma and that high levels of this transcription factor are associated with poor clinical outcome in this disease. Furthermore, TFAP4 and MYCN co-operatively regulate a defined subset of genes to drive cell proliferation and migration in MYCN-driven neuroblastoma cells.

The present study identified 15 genes with more than two-fold changes in gene expression profiling that appear to be regulated by MYCN through TFAP4 or commonly regulated by both transcription factors. Notably, TFAP4 did not regulate any genes in an antagonistic direction to MYCN in this subset of genes, suggesting that these two transcription factors cooperatively regulate specific target genes that play critical roles in the development of the aggressive phenotype of neuroblastoma. This notion was supported by the observation that MYCN and TFAP4 were able to bind to their respective binding sites closely located on the promoters of the two target genes, *PRPS2* and *SDC1* (CD138); while simultaneous depletion of *MYCN* and *TFAP4* resulted in further reduction of promoter activity of both these genes. PRPS2, a key rate-limiting enzyme within the nucleotide biosynthesis pathway, has been previously reported to be a MYC target gene [16]. In cancer cells, PRPS2 has been reported to be involved in nucleotide metabolism in melanoma and lymphoma and hence is proposed to be a potential therapeutic target for MYC-overexpressing cancers [16, 17]. SDC1, a transmembrane (type I) heparan sulfate proteoglycan is involved in growth factor signalling, cell proliferation, migration and cell-matrix interactions via its receptor for extracellular matrix proteins [20]. Ishikawa *et al,* (2010) reported that suppression of SDC1 leads to more invasive and metastatic tumor cells [21]. The molecule can exist in either a membrane-bound or soluble form, and the former cell-surface SDC1 has been shown to have roles in tumor formation and progression [22], while shed SDC1 correlates with a more aggressive phenotype and may be a potential prognostic marker for a number of adult cancers including colorectal cancer, lung cancer and multiple myeloma [18].

*SLC7A6*, the only other gene found to be positively regulated by both MYCN and TFAP4, is a sodium dependent neutral amino acid transporter belonging to the L-type amino acid transporter (LAT) family, and increased expression of some LAT family members has been shown to be critical for control of protein translation in cancer via the mTORC1 pathway [23]. Our findings that high levels of expression of *PRPS2*, *SDC1* and also *SLC7A6* are associated with poor clinical outcome in neuroblastoma, and that PRPS2 and SDC1 are important in proliferation, suggest that these genes may facilitate tumor progression in MYC- and MYCN-driven cancers.

TFAP4 has been shown to repress or activate different target genes, and it is not able to form heterodimers with other helix-loop-helix transcription factors [5]. In our study, we found that TFAP4 was involved more often in repression (9.45%) than activation (3.06%) of MYCN target genes. Similarly, ChIP-sequencing analysis in colorectal cancer cells showed that ChIP signals from TFAP4-repressed genes were more pronounced than those of TFAP4-activated genes [7]. These data suggest that TFAP4 has a major role in gene repression in tumors with deregulated MYC. One of the nine genes we confirmed to be repressed by both MYCN and TFAP4, *CYLD,* a deubiquitinating enzyme that negatively regulates NF-κB activation [24], is a well-established tumor suppressor gene, and loss of *CYLD* expression has been observed in various human cancer types [25]. Our findings that high *CYLD* levels are predictive for good prognosis in neuroblastoma (Supplementary Figure S4) are in agreement with a recent report showing that higher *CYLD* expression in neuroblastoma patient samples correlated with better survival and early tumor stages, and *CYLD* expression was significantly lower in *MYCN*-amplified tumors [26]. In addition to *CYLD*, knockdown of *DENND5A* in MDCKII cells was reported to lead to an increase in cell migration [27], while *SSU72* was shown to be downregulated in the progression to hormone-refractory prostate cancer [28]. In gastric cancer cells, COPS8 has been reported to be part of G protein-coupled receptor pathway responsible for inhibiting the activation of NF-κB [29]. In contrast to these results however, overexpression of *FMR1*, which encodes fragile X mental retardation protein, has been associated with aggressiveness of breast cancer while its inhibition led to a reduction in invasiveness [30]. Interestingly, some of the highest levels of FMR1 are found in differentiated neurons and Gessert *et al*, [31] have shown that this RNA binding protein is necessary for proper neural crest development (the cell of origin of neuroblastoma) in *Xenopus laevis*. Thus, it remains to be established what role FMR1 has in neuroblastoma tumorigenesis. The remaining genes found to be negatively regulated by MYCN and TFAP4, appear to be largely uncharacterised in cancer and their functions in neuroblastoma require investigation.

In colorectal cancer, TFAP4 was found to be required for MYC-induced EMT, migration, and invasion providing strong evidence for this transcription factor being a new regulator of EMT contributing directly to colorectal cancer metastasis [7]. Although MYC can bind to the promoter of *SNAIL,* a transcription factor known to induce EMT [32], induction of *SNAIL* by MYC is largely mediated via upregulation of TFAP4 [7]. In addition, these authors found that SNAIL is also a target of TFAP4. Interestingly, in melanoma cells, SNAIL has been shown to inhibit expression of CYLD, which in turn leads to increased melanoma proliferation and invasion [33]. Taken together with our study, TFAP4 appears to be initially upregulated by MYC or MYCN, after which it cooperatively regulates the same target genes to maximize rapid transcriptional activity in cancer cells. In this regard, a genome-wide analysis of TFAP4 DNA binding using ChIP-sequencing revealed a similar overall gene distribution pattern for TFAP4- and MYC-binding sites in colorectal cancer [7], suggesting that TFAP4 could be involved in regulation of a large number of genes targeted by MYC in this malignancy, and further supporting the concept of TFAP4 and MYC cooperatively regulating a number of genes in MYC-driven cancers.

In MYC–induced EMT in colorectal cancer, TFAP4 has been shown to regulate a number of EMT-associated genes such as *CDH1*, *CDH2*, *VIM* and *SNAIL*. TFAP4 transcriptionally induces *SNAIL*, and both TFAP4 and SNAIL repress *CDH1*. In addition, TFAP4 protein is associated with Vimentin expression and inversely correlated with E-cadherin expression in seven colorectal cancer cell lines [7]. Consistent with this study, we found upregulation of *SNAIL* as well as downregulation of *KRT8* by elevated expression of TFAP4 in SH-SY5Y cells (Supplementary Figure S5). However, *CDH1* was also upregulated in SH-SY5Y cells as well as in SK-N-FI cells, which is not in agreement with the general finding that *CDH1* is repressed in EMT. As an early event in EMT, cells commonly undergo a switch from E-cadherin to N-cadherin, and N-cadherin expression has been associated with increased motility and invasiveness [34]. In our study, *CDH2* remained unchanged following overexpression of *TFAP4*. In addition, we observed no change in expression of *TWIST*, a well-established EMT-inducing gene that promotes tumor invasion and metastasis [35]. Furthermore, we saw reduced expression of *ZEB1*, another master EMT-inducing gene, which is generally activated in EMT [36]. Importantly, we found no change in gene expression of *Vimentin*, a hallmark in cells undergoing EMT (high Vimentin and low E-cadherin expression) [37]. A number of other genes involved in EMT in neuroblastoma, such as *ACTN4*, *MYH9*, *ROCK1* and *TCF3* [38, 39], failed to show any significant changes in our studies. Finally, we did not observe obvious cell morphological changes 72 hours after transient overexpression of *TFAP4*.

Collectively, the present data do not suggest TFAP4 has a major role in the EMT phenotype of neuroblastoma. We showed TFAP4 promotes cell migration in neuroblastoma cell lines BE(2)-C, SH-SY5Y and SK-N-FI (Figure 3, Supplementary Figure S2A) and we believe this is, at least partly, achieved through activation of TFAP4 target genes such as *SDC1*. However, since transient expression of TFAP4 caused upregulation of *SNAIL* and downregulation of *KRT8* in SH-SY5Y cells, and a trend in reduction of *CDH1* at 48 hours compared to 24 hours (still higher than control) in both SH-SY5Y and SK-N-FI cells (Supplementary Figure S5), it could be possible that the effect of TFAP4 on EMT happens at much later timepoints, and this remains to be explored.

MYC is a “master regulator” of two interrelated processes in transformed cells, cellular growth and metabolism, and remodelling of cancer metabolic pathways by MYC is vital for maintenance of a rapid cellular proliferation phenotype in MYC-transformed cells [40, 41]. To our knowledge, this is the first study to investigate the role of TFAP4 in MYCN-driven neuroblastoma. Our data support the existence of a regulatory circuit between MYCN and TFAP4, where TFAP4 is directly induced by MYCN, then cooperates with MYCN to regulate a subset of MYCN-target genes involved in cancer cell proliferation and metastasis, nucleotide and protein synthesis and growth. In particular, we have identified *PRPS2* and *SDC1* as genes that are positively regulated by both MYCN and TFAP4, and which represent novel candidate therapeutic targets for MYCN-driven neuroblastoma.

## MATERIALS AND METHODS

### Gene silencing, transfection, cellular assays

Human neuroblastoma cell lines BE(2)-C and SH-EP were obtained from the laboratory of Barbara Spengler [42]. Human SH-EP/TET21/N cells, derived from SH-EP neuroblastoma cells, express MYCN under the control of tetracycline (Tet-off) [43, 44]. SH-EP/S1 cells were derived from SH-EP cells by stable transfection with *MYCN*-expression vector. Neuroblastoma cell lines IMR-32, SH-SY5Y, SK-N-FI and CHP-134 were obtained from American Type Culture Collection (Rockville, MD). Cell lines were systematically validated using short tandem repeat genetic profiling (CellBank Australia, Sydney) and mycoplasma tested. Cell lines were cultured in DMEM or RPMI containing 10% fetal calf serum (FCS).

Lipofectamine^®^ RNAiMAX and Lipofectamine 2000 (Invitrogen) were used to deliver siRNA (20–40 nM) or plasmids (5 μg *AP4*-*FLAG* (OriGene, MD), MYCN (#240081, Agilent Technologies) or empty vector). Qiagen siRNAs were: Control (#SI03650318); *MYCN* (#SI03078222,#SI03087518); *TFAP4* (#SI00049322, #SI03057558); *SDC1* (#SI00020601, #SI03072230), *PRPS2* (#SI00042903, #SI03076997). SMARTpool human *MYCN* siRNA was from Dharmacon (#M-003913-01).

Colony forming assays were performed as described [45], with cells replated 48 h post-transfection and colonies of greater than 50 cells counted. For cell cycle analysis, cells at 48 h or 72 h post-transfection were fixed with ice-cold 70% ethanol for 1 h before incubation at 37°C (30 min) in PBS with added propidium iodide (50 μg/mL) and RNase A (2 μg/mL). Stained cells were analyzed for DNA content on a FACSCalibur flow cytometer using CELLQuest analysis software (BD Biosciences).

Wound closure assays and transwell migration assays were performed as published [46, 47].

### Western blot

Western blots were performed according to standard procedures [48] using 25 μg of whole-cell extract. Syndecan-1 (SDC1) protein samples were prepared by scraping cells in lysis buffer. Antibodies used were MYCN (B8.4B, #sc-53993, 1:2000, or C-19, #sc-791, 1:500, Santa Cruz) [49], p27^kip1^ (BD Biosciences #610241, 1:2000) [50], TFAP4 (Abcam #ab58288, 1:2000), FLAG (anti-Flag-M2, Agilent Technologies #200471, 1:2000), SDC1 (D4Y7H, Cell Signaling #12922S, 1:1000), PRPS2 (Abnova #H00005634-A01, 1:1000) [16], GAPDH (G-9, Santa Cruz #sc-365062, 1:3000), Actin (Sigma-Aldrich #A2066, 1:5000).

### Microarray analysis

Neuroblastoma BE(2)-C cells were transfected with scrambled control siRNA or two independent siRNAs for *MYCN* and *TFAP4*. 30 h post-transfection, total RNA was extracted with RNeasy Plus Mini kit (Qiagen). Differential gene expression was examined with GeneChip^®^ Human Genome U133 Plus 2.0 Array (Affymetrix). Results were loaded into R package and analysed with BioConductor software.

### Real-time quantitative PCR and Taqman Low Density Array (TLDA) analyses

RNA was extracted using an RNA Extraction kit (Qiagen) and reverse transcribed with MMLV reverse transcriptase (Life Technologies). Gene expression was determined by quantitative PCR (qPCR) using the ABI7900HT sequence detection systems (Applied Biosystems, ThermoFisher Scientific) for TaqMan^®^ Assays, or CFX96 real-time PCR detection system (Bio-Rad) for SYBR^®^ Green assays. The ΔΔCt method was used to compare expression of target genes normalized to the expression of reference genes (*GUSB*, *HPRT* or *actin*).

Gene expression analyses were performed on 208 primary neuroblastoma tumors (COG cohort) from patients enrolled in Children's Oncology Group (COG) Neuroblastoma Biology Study 9047 [46]. qPCR with TaqMan^®^ gene expression assays were conducted on a TLDA platform using a 7900HT Fast Real-Time PCR system (Applied Biosystems, ThermoFisher Scientific). TLDA cards were loaded with 125ng cDNA (RNA equivalent) per loading port. Gene expression levels were determined using the ΔΔCt method relative to a calibrator [48]. Expression values were calculated as the geometric mean of values normalized to control genes *HPRT*, *GUSB* and *PPIA.*

TaqMan^®^ Assays for qPCR were: *TFAP4* (Hs00231478_m1), *MYCN* (Hs00232074_m1); *SDC1* (Hs00896423_m1); *PRPS2* (Hs00267624_m1); *SLC7A6* (Hs00938056_m1); *CYLD* (Hs00211000_m1); *MFSD6* (Hs00214462_m1); *FMR1* (Hs00924547_m1); *C SGALNACT2* (Hs00603821_m1); *DENND5B* (Hs00958915_m1); *SSU72* (Hs00982637_m1); *LCORL* (Hs00766084_m1); *FAM73A* (Hs01594834_m1), *C19orf12* (Hs01107514_m1); *OIP5-AS1* (Hs01587688_g1); *COPS8* (Hs00991301_g1); *HPRT* (Hs02800695_m1); *GUSB* (Hs00939627_m1); *PPIA* (Hs99999904_m1). qPCR assays for *TFAP4* expression using SYBR^®^ Green were performed using published primers [6]. qPCR assays for EMT-associated genes using SYBR^®^ Green were performed using primers listed in Supplementary Table S2.

### Chromatin immunoprecipitation (ChIP) and luciferase assays

ChIP assays were performed as described [43] on neuroblastoma BE(2)-C and SH-EP/TET21/N cells. Antibodies employed for ChIP assays were: MYCN (#sc-53993, Santa Cruz Biotechnology) [49], MAX (#sc-197, Santa Cruz) [51] and TFAP4 (#HPA001912, Sigma-Aldrich) [7]. Primers for quantitative ChIP are listed in Supplementary Table S1. Luciferase assays were performed as published [43].

### Statistical analysis

For molecular and cellular assays, differences between two groups were analyzed by two-tailed Student's *t*-test. *P* < 0.05 was considered statistically significant. Associations between expression levels of a given gene and clinical characteristics of patients were analyzed using Fisher's exact test. Univariate and multivariate analyses for event-free survival (EFS) and overall survival (OS) were performed using SPSS version 22 (IBM, Mainz, Germany) as described [46]. For each cutoff point, a Cox model produced a *P*-value and hazard ratio and the optimal expression cutoff point was determined as described previously [52]. Pearson correlation test was performed to analyze the correlation between expression of *TFAP4* and *MYCN*.

## SUPPLEMENTARY MATERIALS



## Abbreviations

ChIP: chromatin immunoprecipitation
COG: Children's Oncology Group
CYLD: cylindromatosis
EFS: event-free survival
EMT: epithelial-mesenchymal transition
OS: overall survival
PRPS2: phosphoribosyl-pyrophosphate synthetase 2
SDC1: syndecan-1
TFAP4: transcription factor activating enhancer binding protein-4.
