# Supplementary material for: MYCN promotes neuroblastoma malignancy by establishing a regulatory circuit with transcription factor AP4
Source: Oncotarget. 2016 Jul 19;7(34):54937–51. doi: 10.18632/oncotarget.10709 (PMC5342392; doi:10.18632/oncotarget.10709)
Supplement: Supplementary file 1 [file oncotarget-07-54937-s001.pdf]

MYCN promotes neuroblastoma malignancy by establishing a regulatory circuit with transcription factor AP4

Supplementary Materials

Supplementary Table S1: Primers used for quantitative ChIP

SDC1 -1000 For	AAGAGGAACACTGGACACTTC
SDC1 -1000 Rev	GGCAGGCAGGAGAGATGG
SDC1 -500 For	TGAAGAGAGAGGGCGTTG
SDC1 -500 Rev	CCACAAAGTGCTGGAGAC
SDC1 -TSS For	GGAGGAGCGGGAGCCAAG
SDC1 -TSS Rev	GCCCTTCCTTAGCCGTTGC
SDC1 +500 For	CCGAGACCAGGGCTCCAG
SDC1 +500 Rev	GGGCTCCGACAGATGTGG
SDC1 +1000 For	GCTCGGCTTTGTGCTGAAG
SDC1 +1000 Rev	CGCTGAAGGTGGATGCTG
SDC1 +1500 For	AGTCTGCCGCCCGAGTTC
SDC1 +1500 Rev	CGAGACACCGCCACTTCC
SDC1 Dist for	GGGGCCGAATCAGTGTGTAA
SDC1 Dist rev	CCGGGGTGATCTACCAAAGG
PRPS2 -1000 For	CTTGGTTTGTGCCCTGAAGTTGG
PRPS2 -1000 Rev	CCTTCTGCCTGTTGGGTGCTC
PRPS2 -500 For	AAGGCGGTGTCTGCTATCATC
PRPS2 -500 Rev	GTGTGGGCGACTGGTGTTC
PRPS2 -TSS For	CTTCGGGCTTCAGGACCAC
PRPS2 -TSS Rev	GCGGCGGCTAGATGTAGG
PRPS2 +500 For	ACAGACCGTCGGGGAGAGAG
PRPS2 +500 Rev	GGACCAGCCAACCCAGGAC
PRPS2 +1000 For	GCTTCCCTGGCTTCTCCTG
PRPS2 +1000 Rev	ACACAGCAGAGTCCTAGAACAAG
PRPS2 Dist for	AGCCCTCTTCAACTGCTCAC
PRPS2 Dist rev	ATAGTGGAGCCAAGCAGCTC

Supplementary Table S2: Sequence of primers used in qPCR analysis of EMT-associated genes

	Forward	Reverse
MYH9	TCTATGCCATCACAGACACCG	CTTGCCAGCTCCAGATTCAC
ACTN4	CTACCATGCCTTTTCAGGAGC	AGGTGCTCGTTCTCTTGGTTG
VIMENTIN	TCTGGCACGTCTTGACCTTG	TCCTGGATTTCCTCTTCGTGG
TWIST1	TTCTCGGTCTGGAGGATGGA	AATGACATCTAGGTCTCCGGC
TCF3	CTCGGTCATCCTGAACTTGG	TCTCCAACCACACCTGACAC
GUSB	TGGTGCGTAGGGACAAGAAC	CCAAGGATTTGGTGTGAGCG
ROCK1	GAAATGGAGCAGAAGTGCAG	CTCCTTCTCAATCTGAGACACTG
KRT8	AGACAAGGTACGGTTCCTGGAG	ATGTTGCTTCGAGCCGTCTT
ZEB1	CCAGCCAAATGGAAATCAGGATG	GCATTTTCTTTTTGGGCGGTG
SNAIL	ACTATGCCGCGCTCTTTCCT	GCTGCTGGAAGGTAAACTCTGG
CDH1	CGTCCTGGGCAGAGTGAATTT	ACCATCTGTGCCCACTTTGA
CDH2	TCCAGACCCCAATTCAATTAATATTAC	AAAATCACCATTAAGCCGAGTGA

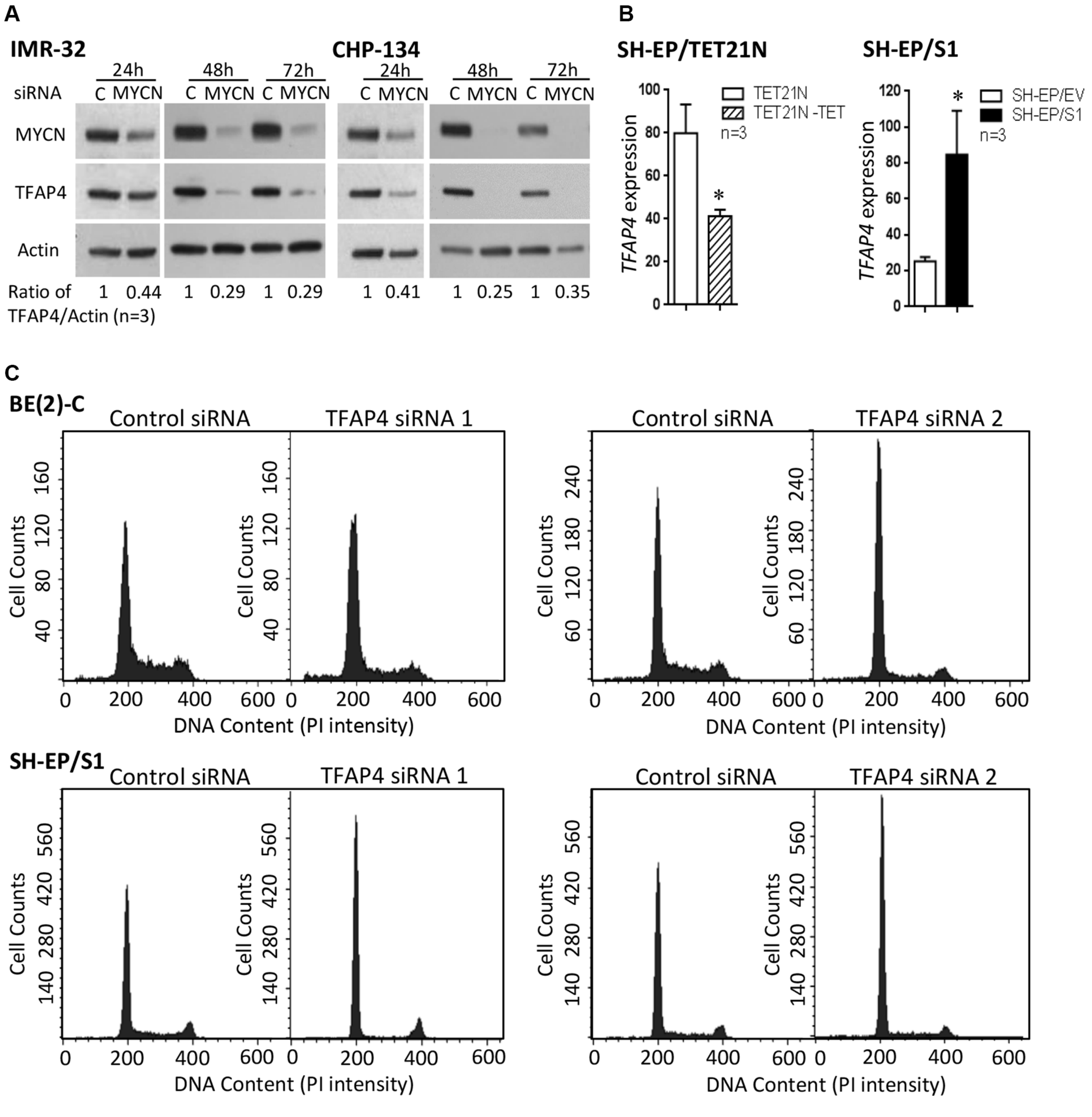

Supplementary Figure S1: (A) Western blots showing TFAP4 expression is downregulated after knockdown of *MYCN* with siRNA in *MYCN*-amplified neuroblastoma IMR-32 and CHP-134 cells. (**B**) qPCR analysis of *TFAP4* gene expression in SH-EP/TET21/N cells 24 hours after TET treatment and SH-EP/S1 cells, respectively. *$P < 0.05$. (**C**) Representative flow cytometry histograms. *TFAP4* depletion leads to cell cycle arrest at G1/S phase 48 hours post siRNA transfection in BE(2)-C and SH-EP/S1 cells.

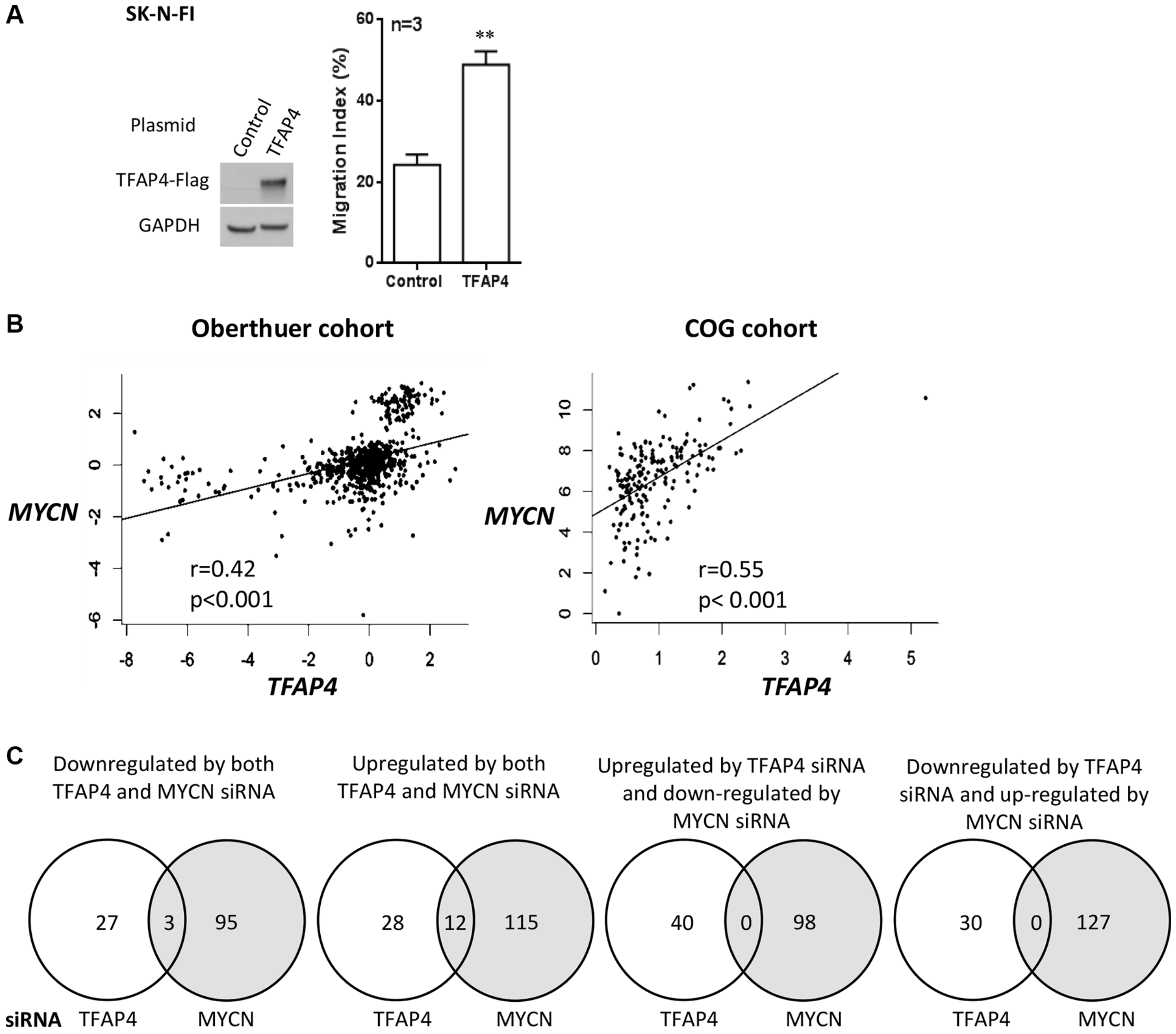

Supplementary Figure S2: (A) Overexpression of *TFAP4* in *MYCN*-non-amplified neuroblastoma SK-N-FI cells increased migration. Mean ± SE ($n = 3$). $^{**}P < 0.01$. Western blot shows overexpression of TFAP4 protein. (**B**) *TFAP4* expression was positively correlated with *MYCN* expression in both Oberthuer cohort and COG cohort patients' datasets. (**C**) Microarray analyses of gene expression in BE(2)-C cells 30 hours after depletion of *MYCN* or *TFAP4*. Differential expression was defined by ≥ 2-fold change when compared to control siRNA group. Common genes regulated by two *MYCN* siRNAs were compared with genes commonly regulated by two *TFAP4* siRNAs.

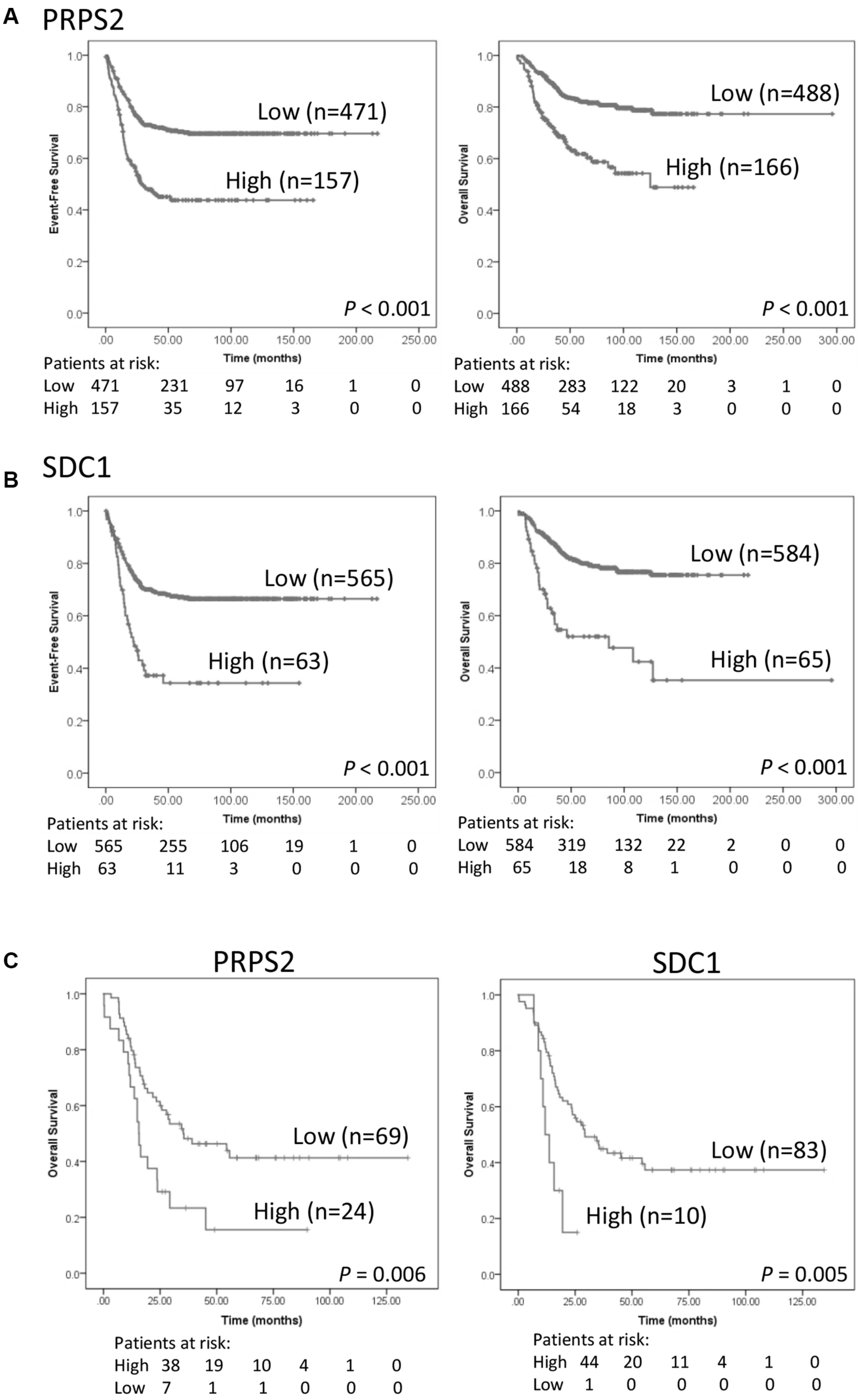

Supplementary Figure S3: (A) Kaplan–Meier curves for EFS and OS for *PRPS2*, dichotomized around the upper quartile (Oberthuer cohort). EFS, Hazard ratio [HR] = 2.32, 95% confidence interval [CI] = 1.76–3.06; $P < 0.001$) and OS, HR = 2.69, 95% CI = 1.92–3.76, $P < 0.001$. (**B**) Kaplan–Meier curves for EFS and OS for *SDC1*, dichotomized around the upper decile (Oberthuer cohort). EFS, HR = 2.46, 95% CI = 1.74–3.49, $P < 0.001$), and OS, HR = 3.4, 95% CI = 2.28–5.06, $P < 0.001$. (**C**) Prognostic significance in the subset of patients with *MYCN*-amplified tumors. Kaplan–Meier curves for OS for *PRPS2* and *SDC1*, dichotomized around the upper quartile and upper decile, respectively (Oberthuer cohort).

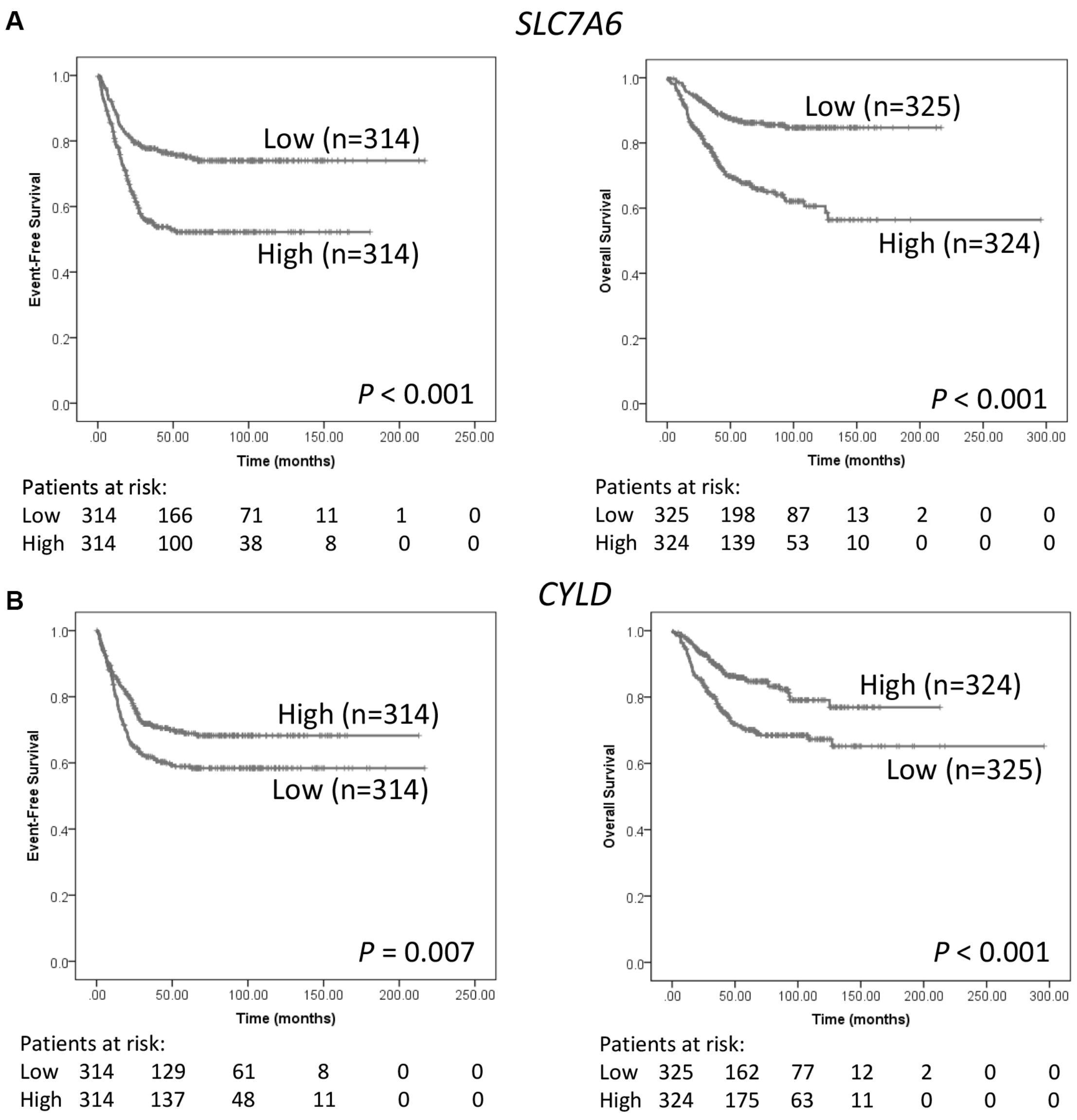

Supplementary Figure S4: Prognostic values of *SLC7A6* and *CYLD* gene expression in 649 primary neuroblastomas (Oberthuer cohort) using median values as cut-off points. (**A**) Kaplan–Meier curves for EFS and OS for *SLC7A6*. EFS, HR = 2.14, 95% CI = 1.63 to 2.83, P < 0.001) and OS, HR = 2.87, 95% CI = 2.00 to 4.12, *P* < 0.001. (**B**) Kaplan–Meier curves for EFS and OS for *CYLD*. EFS, HR = 0.66, 95% CI = 0.51 to 0.87, *P* = 0.007 and OS, HR = 0.51, 95% CI = 0.36 to 0.72, *P* < 0.001.

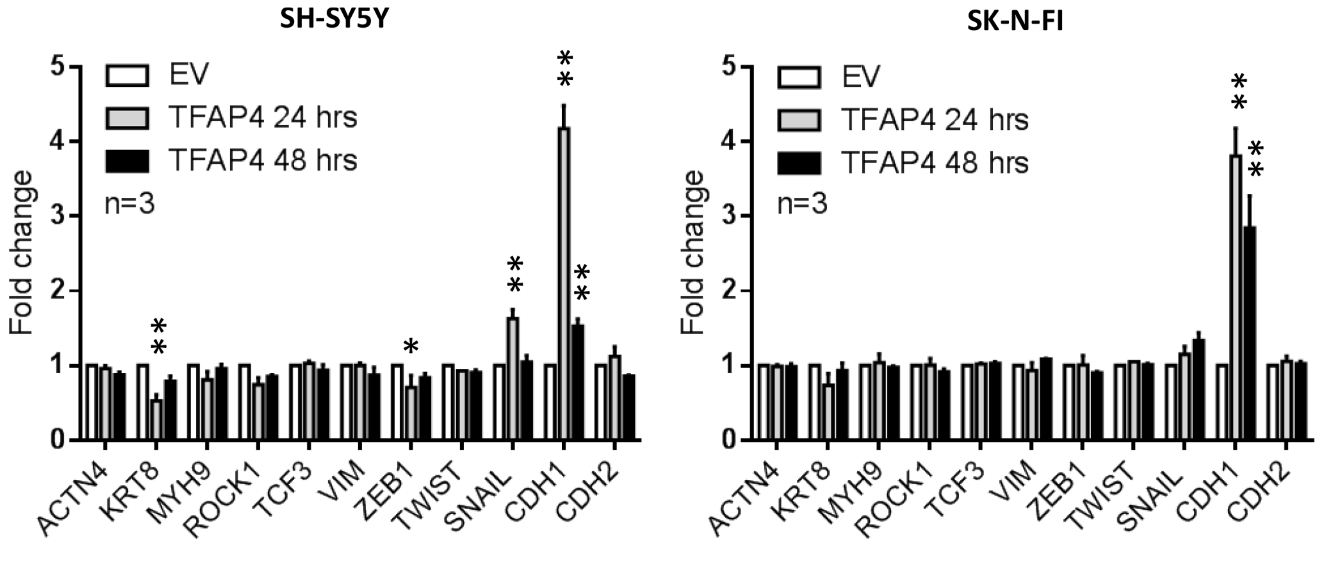

Supplementary Figure S5: qPCR analyses of *EMT*-associated gene expression 24 and 48 hours after transient overexpression of *TFAP4* or empty vector (EV) in neuroblastoma SH-SY5Y and SK-N-FI cells. *$P < 0.05$, **$P < 0.01$.
